# Longitudinal analysis of usage and public awareness of tyrosine kinase inhibitors for CML

**DOI:** 10.3389/fonc.2025.1711453

**Published:** 2025-11-17

**Authors:** Adrian E. Schroer, Tiffany Javadi, Ankit Mehta, Furkan Torlak, Joseph R. McFarland, Girish Kumar, Alexandre L. da Silva, Jamaal Benjamin, Moaz M. Choudhary, Akram Sadeghi, Philipp le Coutre, Michael J. Blaha, Philipp Berning, Omar Dzaye

**Affiliations:** 1 Division of Vascular and Interventional Radiology, Department of Radiology, University of Texas Southwestern Medical Center, Dallas, TX, United States; 2 Johns Hopkins Ciccarone Center for the Prevention of Cardiovascular Disease, Johns Hopkins University School of Medicine, Baltimore, MD, United States; 3 Charité – Universitätsmedizin Berlin, Corporate Member of Freie Universität Berlin and Humboldt-Universität zu Berlin, Berlin, Germany; 4 Department of Pathology and Laboratory Medicine, Emory University School of Medicine, Atlanta, GA, United States; 5 Department of Medicine, Hematology and Oncology, Charité – Universitätsmedizin Berlin, Corporate Member of Freie Universität Berlin and Humboldt-Universität zu Berlin, Berlin, Germany

**Keywords:** tyrosine kinase inhibitor usage, CML treatment, public awareness, infodemiology, Google Trends

## Abstract

**Introduction:**

The treatment landscape of chronic myeloid leukemia (CML) has evolved with the introduction of second- and third-generation tyrosine kinase inhibitors (TKIs), oCering potential advantages over imatinib. We analyzed prescription trends and public awareness of TKIs to assess the adoption of newer agents.

**Methods:**

Monthly US prescription data from the IQVIA National Prescription Audit (NPA) and Google Trends search volumes from March 1, 2017, to November 31, 2024, were analyzed for visual and quantitative trends and correlation patterns. Studied TKIs included imatinib (first generation), dasatinib, bosutinib, nilotinib (second generation), and ponatinib, asciminib (third generation).

**Results:**

Second-generation TKIs increased by 14.4% (15,171 to 17,363 average monthly prescriptions) between 2017 and 2024, while the use of imatinib declined (-10.0%, from 18,704 to 16,835). Bosutinib (+144.1%) and dasatinib (+27.8%) usage increased during this period, while nilotinib prescriptions decreased (-26.0%). Third-generation TKIs saw substantial growth (696 to 2,123 average monthly prescriptions), led by ponatinib (+86.6%) and asciminib (+235.0% since 2022). Online search volumes strongly correlated with prescription trends, particularly for newer TKIs: asciminib (r = 0.85), bosutinib (r = 0.74), nilotinib (r = 0.74), ponatinib (r = 0.63), and dasatinib (r = 0.51). Imatinib showed little correlation (r = 0.21). Prescription patterns varied across disciplines, with Advanced Practice Providers (APPs) prescribing imatinib 9% less frequently than internists/primary care physicians (PCPs).

**Discussion:**

These data highlight a shift toward newer TKIs in CML treatment, mirroring guideline recommendations and rising public awareness. Online search trends complement traditional prescription monitoring, oCering near real-time insights into evolving prescribing practices and drug adoption.

## 1 Introduction

Chronic myeloid leukemia (CML) is a myeloproliferative neoplasm characterized by overproduction of maturing myeloid precursor cells. The hallmark of CML is the Philadelphia (Ph) chromosome, generated by a specific chromosomal translocation between chromosomes 9 and 22 (t(9;22)(q34;q11)), resulting in the *BCR::ABL1* fusion gene. This oncogene encodes the functional *BCR-ABL1* protein, which leads to constitutive activation of tyrosine kinase and promotes growth of CML cells (1, 2). Due to the critical role of *BCR::ABL1* in the pathogenesis of CML, tyrosine kinase inhibitors (TKIs) have become the cornerstone of modern CML therapy (3) with molecular response to TKI treatment serving as the key determinant of long-term prognosis in CML (4). Under TKI therapy, patients have demonstrated 8-year survival rates of 87% (5). Typically, patients remain on TKI treatment for extended periods often spanning several years and often achieving treatment-free remission. Treatment modifications occur primarily in patients who develop resistance to the therapy or experience intolerable side effects (3).

First-line therapy for chronic myeloid leukemia (CML) involves the use of first-generation TKI imatinib (Gleevec) or second-generation TKIs such as bosutinib (Bosulif), nilotinib (Tasigna), and dasatinib (Sprycel) (3). However, patients who do not respond and/or become refractory to one of these agents generally receive treatment with a newer third-generation TKI, including ponatinib (Iclusig) and asciminib (Scemblix). These drugs are of particular importance in the treatment of disease resistance, including patients with *T315I* mutation or for drug intolerance (6, 7), which, given the long course of treatment, occur in a fraction of patients (8–10).

Online search behavior has become a useful means of assessing public health trends, particularly prescription trends, in near real-time (11, 12). Several studies could indicate correlations between online search behavior and public health related topics such as infection outbreaks of influenza or COVID-19 (13, 14), hospital admissions, cardiovascular disease and prescription trends, thus offering an easily accessible means of mirroring prescription trends and in general interest in different drugs (15–18). With more than 90% of all online searches, Google represents the main online search engine. To this end, Google Trends has become the main resource for the analyses of public health-related online search patterns and trends (12). Given the evolving therapeutic landscape of TKI-based treatments for CML, we aimed to evaluate prescription trends and public awareness represented by online searches, as well as prescription behaviors across medical specialties. Additionally, we sought to explore how online searches might reflect actual prescriptions and potentially mirror prescription trends.

## 2 Materials and methods

### 2.1 Drug collection – prescription data

Prescription data were gathered from the IQVIA National Prescription Audit (NPA) database. The NPA provides a measure of overall US national prescription dispensing information from retail, mail-order, and long-term care pharmacies. These data include prescription data from approximately 90% of all outpatient prescription activity in the United States and are then projected to estimate all retail transactions. Dispensed prescriptions are recorded irrespective of the payer type, including both insured and self-pay cases. Further detailed information on the data collection process can be found elsewhere (16, 19, 20). In brief, IQVIA links the NPA to the American Medical Association’s Physician Masterfile and other professional organization records to confirm the primary specialty of prescribers.

We gathered monthly prescription data for the US from March 1, 2017, to November 31, 2024. Total monthly dispensed prescriptions, prescriber specialty, and brand names of the drugs used among all patients were extracted. Our analyses considered total dispensed prescriptions (TRx), which encompass new and refill prescriptions. In this analysis, “prescriptions” hereafter refers to total dispensed prescriptions. For prescriber-related information, physician assistants and nurse practitioners were categorized as advanced practice providers (APP). General practitioners were referred to as primary care physicians (PCP)/internists, including family practice, general practice, general preventive medicine, geriatrics, internal medicine, internal medicine/pediatrics, osteopathic medicine, and pediatrics, as previously reported (20).

### 2.2 Data collection – online search data

We extracted monthly search data using the Google Trends for Health Application Programming Interface (16, 20, 21). Data were retrieved from March 1, 2017, to November 31, 2024. Online search volumes were measured as the number of searches per 10 million Google searches. Search data for following brand and generic names of TKIs were extracted: Imatinib *(Gleevec)*, Bosutinib *(Bosulif)*, Nilotinib *(Tasigna)*, Dasatinib *(Sprycel)*, Ponatinib *(Iclusig)* and Asciminib *(Semblix)*. Hereafter reported TKI search data represent aggregated online search volumes for both brand and generic names to ensure representation. As such, the online search data analyzed in this study represent the combined online searches of generic and brand searches.

### 2.3 Statistical and graphical analysis

All analysis was done using the python programming language version 3.12. Libraries used for data aggregation and statistical analysis, including computation of Spearman’s correlation coefficient, were NumPy, Pandas and SciPy. Data visualization was carried out using Matplotlib and Seaborn.

## 3 Results

### 3.1 Prescription trends for individual first, second- and third-generation TKIs

**Figure 1:**
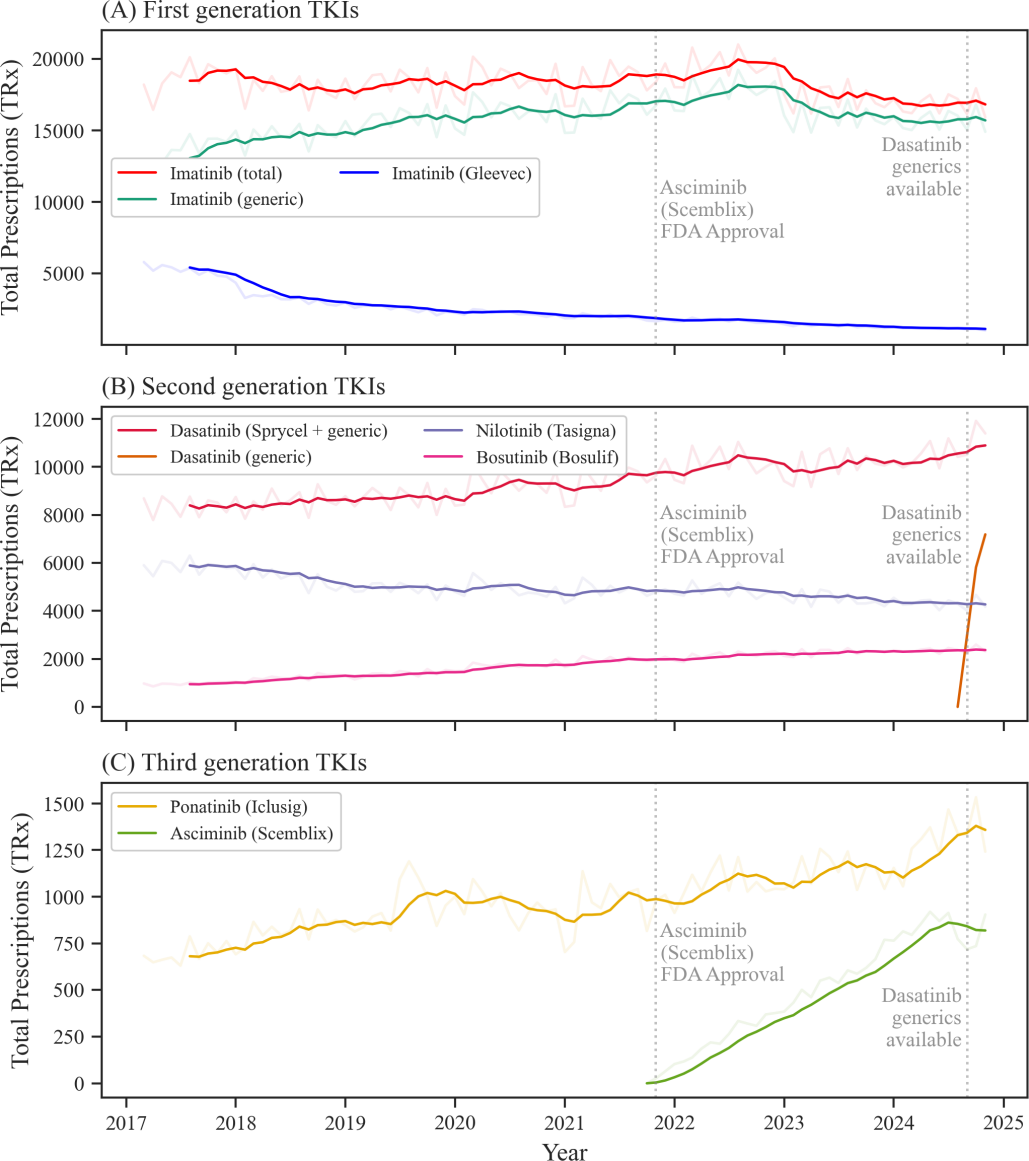
Prescription trends for tyrosine kinase inhibitors (TKIs) used in CML treatment. Prescription data for individual first **(A)**, second **(B)**, and third **(C)** generation TKIs. The blurred-out curves show monthly prescription trends for each drug, and the focused curves show the 6-month moving average (not for generic dasatinib). CML indicates Chronic myeloid leukemia. Source: IQVIA National Prescription Audit (March 2017-November 2024).

During the study period between 03/2017 and 11/2024, imatinib/Gleevec, the first TKI approved by the FDA for the treatment of CML in 2001, showed the highest US prescription volumes with an average of 18,704 combined monthly prescriptions in 2017 (**Figure 1a**, **Supplementary Tables 1**, **2**). Over time, US monthly prescriptions of imatinib decreased to an average of 16,835 in 2024 (**Table 1**). This represented a relative decrease of -10.0% between 2017 and 2024. While Gleevec alone accounted for 5,223 US monthly prescriptions in 2017 (27.9% of total imatinib prescriptions), Gleevec prescriptions decreased to 1,149 in 2024 (6.8% of total imatinib prescriptions).

**Table 1:** Tyrosine kinase inhibitor prescriptions rates over time.

Year	Mean monthly dispensed prescriptions (annual change in %)							
	Imatinib (generic)	Gleevec (branded imatinib)	Dasatinib (generic)	Sprycel (branded dasatinib)	Nilotinib (Tasigna)	Bosutinib (Bosulif)	Ponatinib (Iclusig)	Asciminib (Scemblix)
2017	13481	5223	0	8354	5849	968	696	0
2018	14644 (8.6)	3278 (-37.2)	0	8550 (2.3)	5388 (-7.9)	1207 (24.7)	824 (18.4)	0
2019	15780 (7.8)	2556 (-22.0)	0	8729 (2.1)	4948 (-8.2)	1372 (13.6)	942 (14.3)	0
2020	16310 (3.4)	2230 (-12.8)	0	9251 (6.0)	4918 (-0.6)	1718 (25.2)	954 (1.3)	0
2021	16587 (1.7)	1909 (-14.4)	0	9515 (2.8)	4831 (-1.8)	1945 (13.2)	953 (-0.1)	8^b^
2022	17802 (7.3)	1695 (-11.2)	0	10164 (6.8)	4844 (0.3)	2144 (10.2)	1070 (12.3)	246 (3137.4)
2023	15941 (-10.5)	1340 (-20.9)	0	10060 (-1.0)	4491 (-7.3)	2266 (5.7)	1137 (6.2)	556 (126.6)
2024	15687 (-1.6)	1149 (-14.3)	1450^c^	9222 (-8.3)	4328 (-3.6)	2363 (4.3)	1299 (14.3)	824 (48.1)

a Data for January 2017 – February 2017 and December 2024 not available.

b First asciminib prescriptions were recorded in November 2021.

c First generic dasatinib prescriptions were recorded in September 2024.

Source: IQVIA National Prescription Audit (March 2017-November 2024).

Among newer, second-generation TKIs approved by the FDA from 2006 onward, dasatinib/Sprycel emerged as the most frequently dispensed TKI (**Figure 1b**, **Supplementary Tables 1**, **2**). Between 2017 and 2024, US average monthly prescriptions increased from 8,354 to 10,673, showing a 27.8% growth (**Table 1**). Before its patent expiration in September 2024, Sprycel accounted for all second-generation TKI prescriptions (10,423 US prescriptions in August 2024). Following the availability of generic dasatinib, Sprycel prescriptions declined to 4,209 (-59.6%) by November 2024, while generic prescriptions increased to 7,188. Bosutinib/Bosulif had an average of 968 US monthly prescriptions 2017. However, when compared to other first- and second-generation TKIs, bosutinib showed the highest growth, increasing by 144.1% to 2,363 US monthly dispensed prescriptions in 2024. In contrast, nilotinib prescriptions declined from a monthly average of 5,849 in 2017 to 4,328 in 2024 (-26.0%).

For third-generation TKIs, Ponatinib showed continuously increasing US prescriptions from 2017 to 2024, from 696 average US monthly prescriptions (2017) to 1,299 (2024), representing an 86.6% increase over time (**Figure 1c**, **Supplementary Table 1**). For Asciminib, first US prescriptions were recorded in 11/2021 following the FDA approval for CML treatment in 10/2021 (22). In 2022, US average monthly prescriptions reached 246, which increased to 824 (+235.0%) for 2024.

Next, we analyzed prescription trends across TKI generations, as shown in **Figure 1**. Imatinib (Gleevec) was the only first-generation TKI, serving as the sole representative in the dataset. Second-generation TKIs saw a 14.4% increase, from 15,171 US monthly prescriptions in 2017 to 17,363 in 2024 (**Supplementary Table 3**). In contrast, third-generation TKIs had lower prescription volumes but experienced strong growth (+204.9%), rising from 696 US average monthly prescriptions in 2017 to 2,123 in 2024.

### 3.2 Prescription trends across specialties

**Figure 2:**
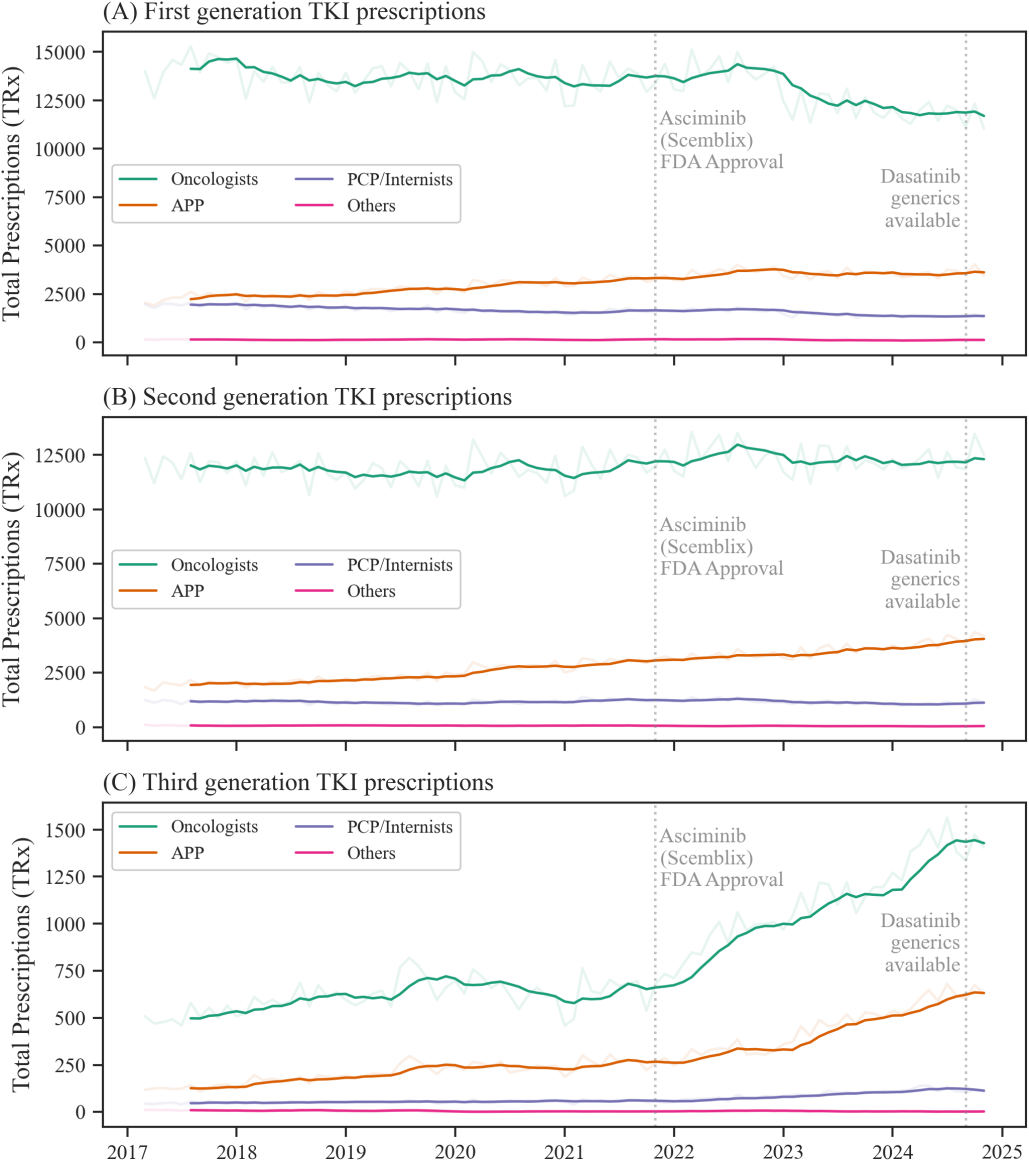
Prescription trends for tyrosine kinase inhibitor (TKI) generations across top prescribing specialties. Prescription data for first **(A)**, second **(B)**, and third **(C)** generation TKIs for each top prescribing discipline. The blurred-out curves show monthly prescription trends for each prescribing discipline, and the focused curves show the 6-month moving average. APP indicates advanced practice providers, PCP, primary care physicians. Source: IQVIA National Prescription Audit (March 2017-November 2024).

In a next step, the drug choices across the top 3 prescribing specialties in the US, oncologists, APP and PCP/internists were analyzed (**Figure 2**). Across all TKI generations, oncologists were the top prescribing discipline accounting for 73.7% of all TKI prescriptions during the study period, followed by APP (17.5%) and PCPs/internists (8.1%) (**Figure 2**, **Supplementary Table 4**). Other medical disciplines showed a minimal role (< 1%).

For imatinib/first-generation TKIs, oncologists’ average prescription rate in relation to total TKI prescriptions by specialty was 53.4% in 2017, which decreased to 46.3% in 2024 (**Supplementary Figure 2a**, **Supplementary Table 4**). Among APP, imatinib prescription rates decreased from 52.5% to 44.0% (**Supplementary Figure 2b**, **Supplementary Table 4**) during the same time period. For PCP/internists, the proportion of imatinib/first-generation TKI among all TKI was higher when compared to other specialties, accounting for 61.3% of TKIs in 2017 and decreased to 53.0% in 2024 (**Supplementary Figure 2c**, **Supplementary Table 4**).

Second-generation TKIs were used at a rate of 44.7% in 2017 by oncologists, compared to 48.3% in 2024. For APPs, second-generation TKIs represented 44.6% of all TKIs prescriptions in 2017 and reached 48.5% in 2024, while for PCPs/internists, their share increased from 37.2% to 42.4%.

With ponatinib as the only approved third-generation TKI from 2017 to 2020 and asciminib expanding the class after October 29, 2021, third-generation TKIs accounted for 1.9% of total TKI prescriptions in 2017 among oncologists, increasing to 5.5% in 2024. Third-generation TKIs were more frequently prescribed by APPs, with their prescription rate increasing from 2.9% to 7.4% of all TKIs prescribed by APPs. The prescription rate among PCPs/internists also increased from 1.5% to 4.6% during this period.

### 3.3 Online search trends for TKIs

**Figure 3:**
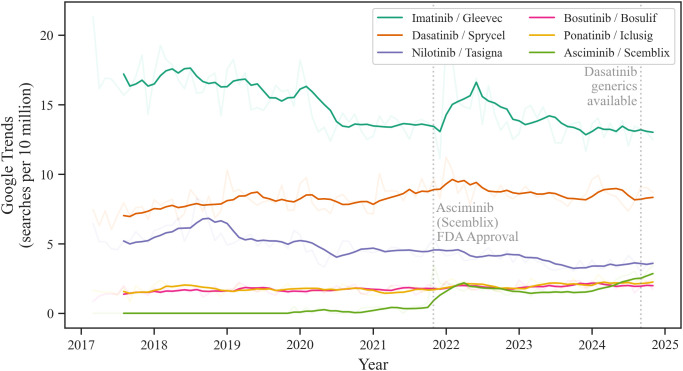
Online searches for tyrosine kinase inhibitors (TKIs). Online search volumes as searches per 10 million searches for aggregated brand and generic names of individual TKIs. The blurred-out curves show monthly online searches for each TKI, and the focused curves show the 6-month moving average. Source: Google Trends (March 2017-November 2024).

Online search trends for TKIs were subsequently analyzed. Search interest for imatinib decreased by 22.2% during the study period to an average of 13.2 per 10 million searches in 2024 (**Figure 3**, **Supplementary Table 5**). Dasatinib was the second most searched TKI after imatinib, with search volumes rising by 21.6% to 8.7 per 10 million searches. In contrast, nilotinib search interest declined by 32.9% to 3.5 per 10 million searches, mirroring its decreasing prescription trends. Online searches for bosutinib, a second-generation TKI, demonstrated a relative increase of 42.0% resulting in 2.0 per 10 million searches in 2024. Similarly, search volumes for ponatinib also surged by 46.7% to 2.3 per 10 million searches. Following its 2021 approval, asciminib saw a significant surge in search interest, reaching an average of 2.5 per 10 million searches in 2024.

Aggregated online search data by TKI generation revealed comparable volumes for first-generation (imatinib) and second-generation TKIs in 2017, with 16.9 and 13.8 per 10 million searches, respectively (**Supplementary Table 6**). Notably, searches for second-generation TKIs surpassed those for first-generation TKIs in 2020, reaching 14.2 per 10 million in 2024, compared to 13.2 for imatinib/Gleevec. Like third-generation TKI prescription trends, search interest increased significantly, rising from 1.5 per 10 million in 2017 to 4.8 in 2024 - a 212.6% increase, aligning with the 204.9% rise in prescriptions.

### 3.4 Correlation of prescriptions and Google Trends

**Figure 4:**
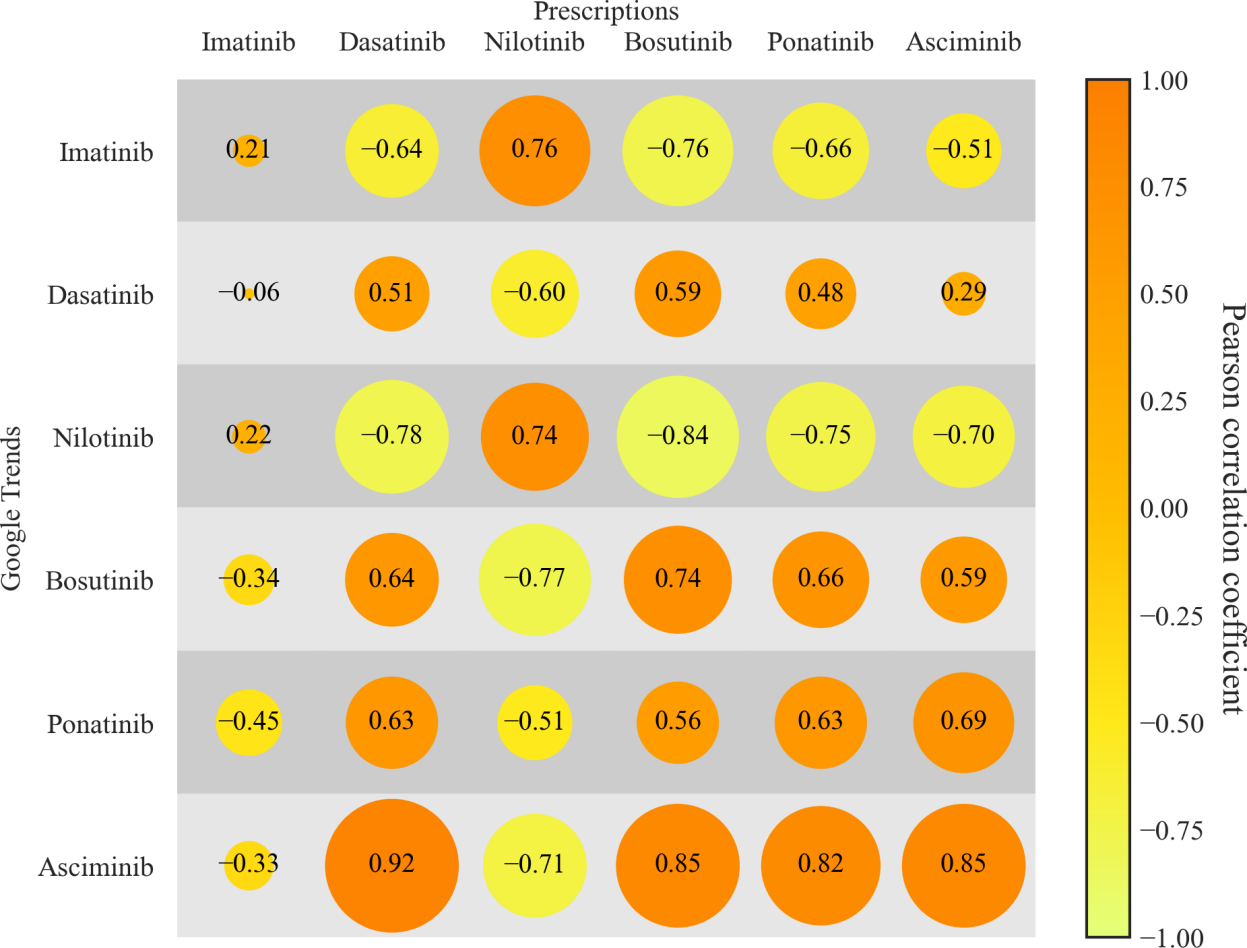
Correlations between TKI prescriptions and online searches. Correlation between TKI-specific prescription data and corresponding online search volumes, expressed as Pearson correlation coefficients (displayed as numbers within the bubbles). Data for prescriptions and online searches were aggregated by quarterly intervals. Orange bubbles represent positive correlations, whereas yellow/green bubbles represent negative correlations. Source: IQVIA National Prescription Audit (March 2017-November 2024).

Ultimately, we analyzed correlation patterns between TKI prescriptions and corresponding online searches. As depicted in **Figure 4**, the correlation matrix shows a size and color-coded representation of the correlation coefficients between quarterly prescription rates and quarterly online searches. For imatinib/first-generation TKIs no correlation (r= 0.21 (95%-CI: -0.15 – 0.52), p = 0.25) between dispensed prescriptions and online searches was observed. For the second-generation TKIs, dasatinib showed a moderate correlation (r = 0.51 (95%-CI: 0.20 – 0.73), p < 0.1). Bosutinib showed a strong positive correlation of 0.74 (95%-CI: 0.53 – 0.86, p < 0.1). Nilotinib, a drug for which prescriptions and search interest decreased over time, showed a correlation coefficient of 0.74 (95%-CI: 0.53 – 0.86, p < 0.1). Asciminib exhibited the strongest correlation between prescription rates and Google trends data, with a correlation of 0.85 (95%-CI: 0.71 – 0.92, p < 0.1). Ponatinib showed a moderate correlation of 0.63 (95%-CI: 0.36 – 0.80, p < 0.1).

## 4 Discussion

The present study provides a comprehensive analysis of US prescription trends and public awareness of TKIs commonly used for the treatment of CML between 2017 and 2024. Our study has four main findings. First, imatinib remained the most frequently prescribed TKI in the US among all other approved TKIs. Second-generation TKI usage has increased substantially, while third-generation TKIs, despite a rapid ongoing overall rise, proportionately remained at approximately 6% of all TKI prescriptions in 2024. Third, oncologists continued to be the main prescribers of TKIs used for CML treatment, while TKI prescriptions by APPs increased by 84% over time. Ultimately, public awareness as represented by online search volumes closely followed prescription trends for bosutinib, nilotinib, asciminib and ponatinib with highest correlations for asciminib.

To the best of our knowledge, this study is the first to comprehensively analyze US drug usage of TKIs, which are primarily approved for the treatment of CML. Consistent with previous reports highlighting the superior efficacy of second-generation TKIs in CML (23–25), imatinib may still be preferred due to its more favorable side effect profile (26). Additionally, as of September 2018 NCCN guidelines have recommended second-generation TKIs as first-line treatment for intermediate- or high-risk patients and younger patients (3, 27). This is in line with our findings, showing overall comparable usage patterns between imatinib/first-generation and second-generation TKIs. Nevertheless, given the higher increase in the proportion of second-generation TKI usage compared to imatinib/first-generation TKIs, this finding may be explained by the potentially higher efficacy of second-generation TKIs (23, 28, 29) as well as changes in guideline recommendations. The availability of generic imatinib helped reduce the cost of CML treatment (30), leading to a clear shift toward generic imatinib over Gleevec. Similarly, our data suggest a rapid transition from Sprycel prescriptions to generic dasatinib after September 2024 following its patent expiration. Thus, the introduction of second-generation generics might further facilitate the adoption of second-generation TKIs in first-line CML therapy. As of January 2024, the US patent for Tasigna has expired; however, no generic versions of nilotinib have been made available in the US at the time of writing. In August 2024, the European Medicines Agency (EMA) approved Nilotinib Accord as the first nilotinib generic (31), raising questions about potential US market entry.

Our analyses additionally revealed that non-physician professionals, such as APPs, including nurse practitioners and physician assistants, accounted for a large proportion of all US TKI prescriptions throughout the study period. Notably, there were no substantial differences in TKI drug (generation) usage between APPs and oncologists, the primary prescribing specialty. Previous studies have shown that APPs play an increasing role in prescribing, particularly in the management of oncology patients, where they are integral to patient care (32, 33). This has as well become evident in the management of patients chronic diseases (33), where APPs manage patient for counseling, drug prescriptions, treatment and follow-up visits (34). Our data further highlight the growing role of APPs in the management of CML patients.

Among APPs, third-generation TKIs were more frequently prescribed when compared to other disciplines, suggesting that APPs seemed to be involved in more complex treatment decisions for patients with relapsed or refractory CML. Overall, oncologists and APPs showed similar prescribing patterns, favoring second- and third-generation TKIs more often than internists, who tended to prescribe imatinib more frequently.

Our Google Trends search analysis revealed a strong correlation for asciminib, the most recently FDA-approved TKI for CML (approved October 29, 2021), consistent with prior studies linking increasing drug usage to higher online search interest (15, 16, 35). Notably, even drugs with declining prescriptions, such as nilotinib, showed strong correlations, suggesting Google Trends can also track downward trends for drugs in CML treatment. These findings highlight Google Trends as a potential near real-time tool to complement traditional prescription analyses. Google Trends can offer avenue for monitoring low-latency situational awareness: spikes in TKI can prompt targeted patient education/clinician outreach and pharmacy planning; regulators and health systems/manufacturers can anticipate short-term demand. Additionally, Google Trends data could further inform public health related research and may aid surveillance of guideline adherence.

Our study has limitations. First, the prescription data were not exclusive to CML patients, as these TKIs are also prescribed for conditions such as acute lymphoblastic leukemia (ALL) and gastrointestinal stromal tumors (GIST). Despite similar U.S. incidences for CML (~2.0/100,000) (36) and ALL (~1.9/100,000) (37), TKIs are used in nearly all CML cases, whereas in ALL they are largely confined to the Philadelphia chromosome–positive subset (38), ~20–30% of (adult) cases (39). GIST is less common (~0.7/100,000) (40), and TKIs are used primarily for unresectable/metastatic disease and as adjuvant therapy in high-risk resected tumors (41). As such, the presented data are expected to be driven mainly by CML, while smaller contributions from Ph-positive ALL and GIST cannot be entirely excluded.

Second, the prescription data from the National Prescription Audit is derived from a sample of outpatient pharmacies and extrapolated to estimate total prescriptions, which may introduce some margin of error. Online search interest may be driven by news coverage, regulatory announcements, guideline changes, and high-profile publications, creating spikes that are not directly tied to prescriptions activity. Such events can also precede or follow changes in prescribing with variable lags. Accordingly, the association between search activity and prescriptions should be interpreted as observational.

While we observed a clear increase in TKI prescriptions by APPs since 2017, we cannot determine whether this trend reflects a CML-specific shift or is part of a broader increase in non-physician prescribing. The physician assistant profession grew by 27.9% between 2019 and 2023 (42), suggesting that the increase in prescriptions may in part reflect general workforce expansion. Therefore, additional research into APP prescription behavior across other medical fields is warranted to better understand and determine whether the observed increase in TKI prescribing is specific to hematology or mirrors broader trends across all specialties. Prescription data analyzed in this study are representative of the US only and thus cannot be projected to other markets.

This study highlights the growing adoption of second- and third-generation TKIs in CML treatment, alongside the increasing role of non-physician professions such as advanced practice providers in prescribing decisions. Additionally, our findings reveal a strong link between TKI prescription trends and public awareness as represented by online searches, underscoring its potential as a real-time tool to for tracking the uptake and changes of approved TKI therapies used for CML treatment.

## Data Availability

The original contributions presented in the study are included in the article/**Supplementary Material**. Further inquiries can be directed to the corresponding author.
