# Supplementary material for: Longitudinal analysis of usage and public awareness of tyrosine kinase inhibitors for CML
Source: Front Oncol. 2025 Nov 17;15:1711453. doi: 10.3389/fonc.2025.1711453 (PMC12665558; doi:10.3389/fonc.2025.1711453)
Supplement: Supplementary file 1 [file DataSheet1.docx]

Supplementary Material

# Supplementary Figures and Tables

## Supplementary Figures

**Supplementary Figure 1. Prescription Trends for Tyrosine kinase inhibitors (TKIs) used in CML treatment by drug generation.**


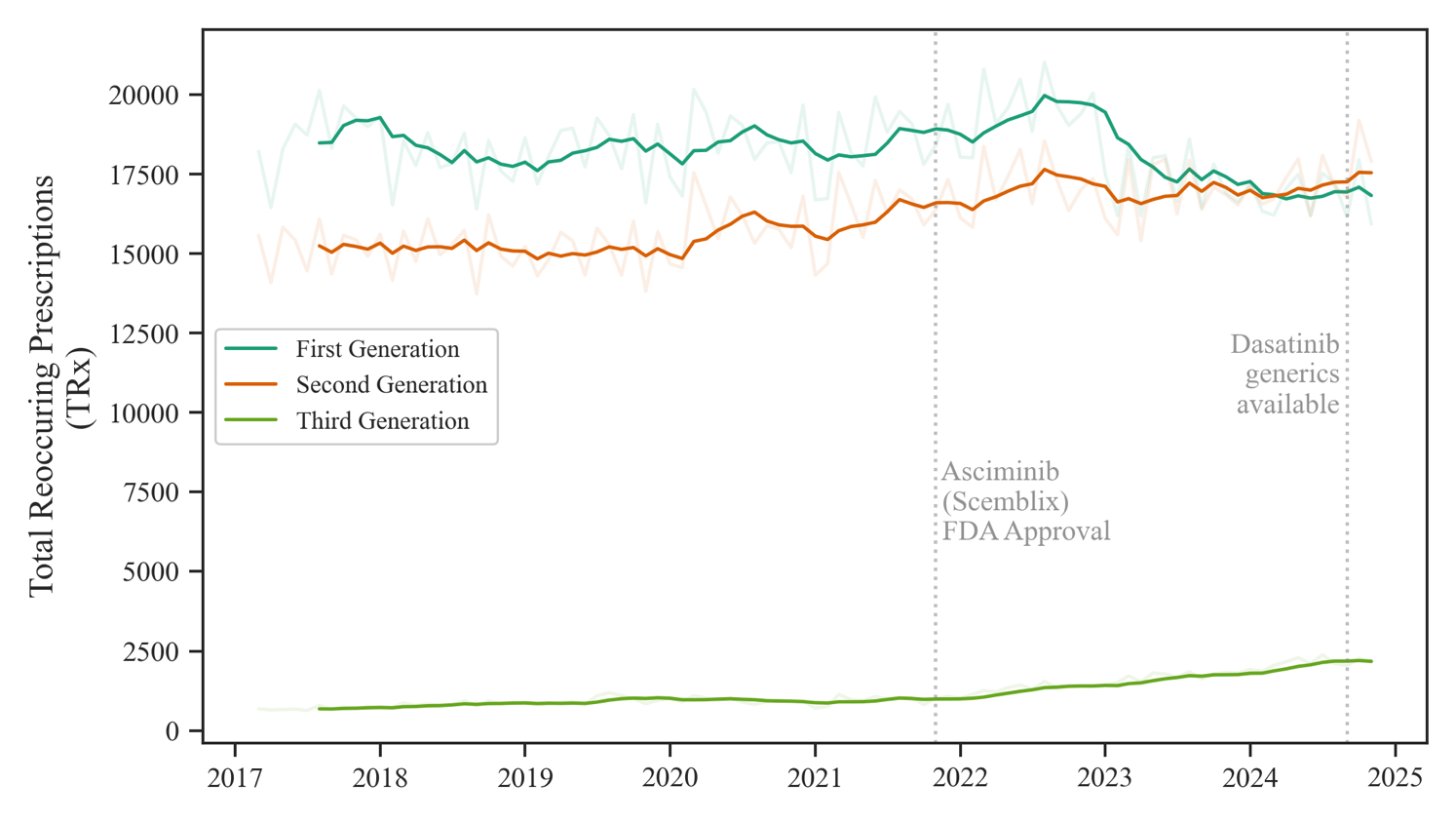


The blurred-out curves show monthly prescription trends for each drug generation, and the focused curves show the 6-month moving average.

**Supplementary Figure 2. Physician drug choice for Tyrosine kinase inhibitors (TKIs) used for CML treatment.**


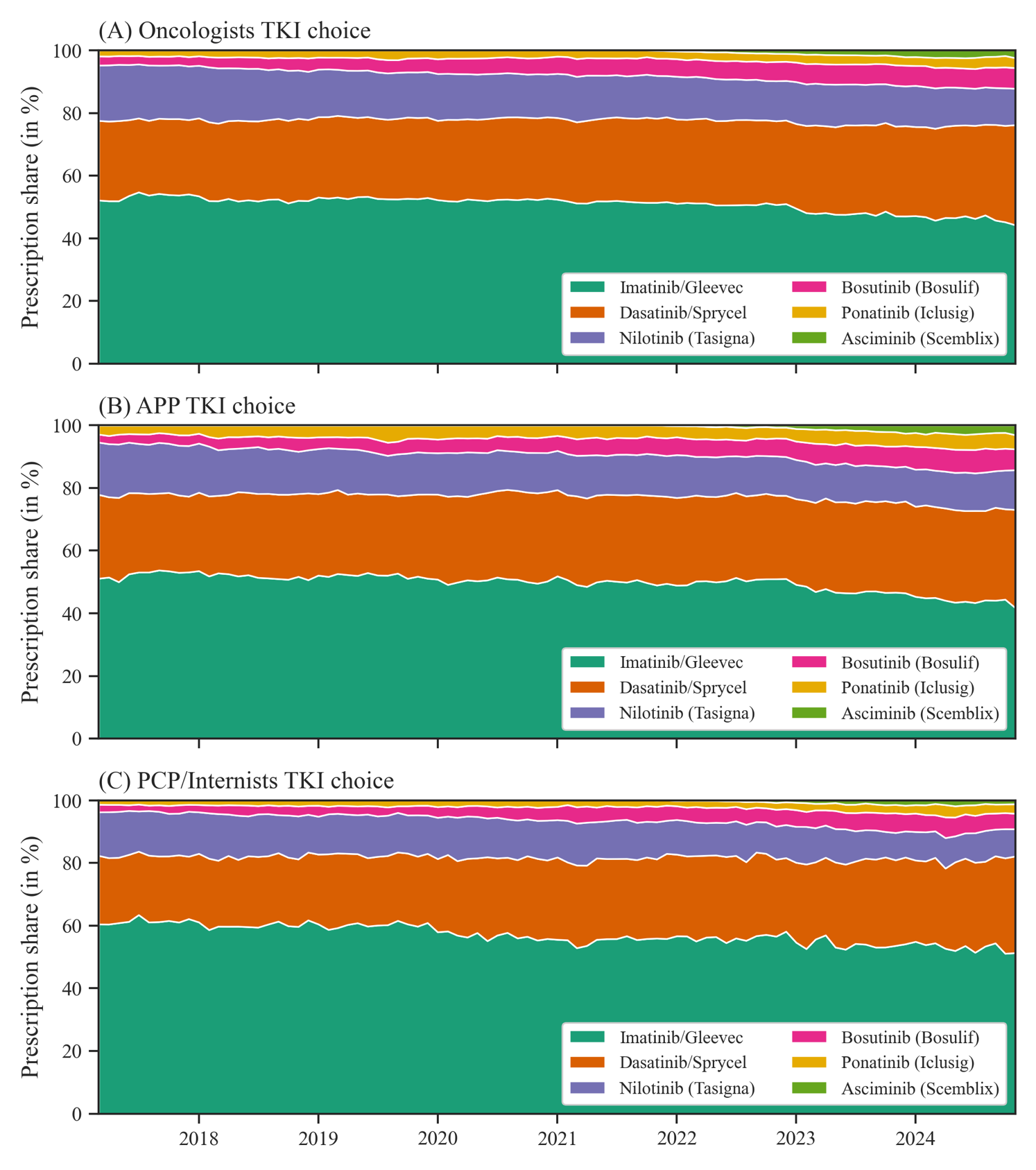


Proportion of total TKI prescriptions attributed to each individual drug by Oncologists (A), APP (B), and PCP/Internists (C).

## Supplementary Tables

**Supplementary Table 1. Monthly Prescriptions of TKIs approved for treatment of CML**

| **date** | **Imatinib Total** | **Imatinib (generic)** | **Gleevec (branded imatinib)** | **Dasatinib Total** | **Dasatinib (generic)** | **Nilotinib** | **Bosutinib** | **Ponatinib** | **Asciminib** |
| --- | --- | --- | --- | --- | --- | --- | --- | --- | --- |
| 2017-03 | 18205 | 12409 | 5796 | 8690 | 0 | 5903 | 974 | 683 | 0 |
| 2017-04 | 16445 | 11254 | 5191 | 7786 | 0 | 5444 | 853 | 648 | 0 |
| 2017-05 | 18288 | 12704 | 5584 | 8774 | 0 | 6084 | 968 | 662 | 0 |
| 2017-06 | 19064 | 13636 | 5428 | 8465 | 0 | 6002 | 955 | 674 | 0 |
| 2017-07 | 18725 | 13611 | 5114 | 7923 | 0 | 5617 | 912 | 630 | 0 |
| 2017-08 | 20123 | 14750 | 5373 | 8764 | 0 | 6306 | 1012 | 787 | 0 |
| 2017-09 | 18303 | 13393 | 4910 | 7906 | 0 | 5510 | 942 | 667 | 0 |
| 2017-10 | 19635 | 14420 | 5215 | 8606 | 0 | 5949 | 1015 | 750 | 0 |
| 2017-11 | 19266 | 14426 | 4840 | 8514 | 0 | 5890 | 1028 | 699 | 0 |
| 2017-12 | 18984 | 14205 | 4779 | 8110 | 0 | 5782 | 1022 | 762 | 0 |
| 2018-01 | 19323 | 14978 | 4345 | 8749 | 0 | 5802 | 1053 | 691 | 0 |
| 2018-02 | 16524 | 13241 | 3283 | 7843 | 0 | 5335 | 982 | 726 | 0 |
| 2018-03 | 18545 | 15063 | 3482 | 8563 | 0 | 5949 | 1192 | 865 | 0 |
| 2018-04 | 17766 | 14368 | 3398 | 8219 | 0 | 5391 | 1146 | 792 | 0 |
| 2018-05 | 18794 | 15296 | 3498 | 9099 | 0 | 5732 | 1260 | 839 | 0 |
| 2018-06 | 17701 | 14508 | 3193 | 8404 | 0 | 5348 | 1203 | 794 | 0 |
| 2018-07 | 17824 | 14640 | 3184 | 8609 | 0 | 5503 | 1170 | 815 | 0 |
| 2018-08 | 18784 | 15460 | 3324 | 8948 | 0 | 5482 | 1301 | 933 | 0 |
| 2018-09 | 16399 | 13533 | 2866 | 7878 | 0 | 4740 | 1112 | 771 | 0 |
| 2018-10 | 18548 | 15370 | 3178 | 9278 | 0 | 5528 | 1408 | 931 | 0 |
| 2018-11 | 17593 | 14758 | 2835 | 8582 | 0 | 5006 | 1327 | 856 | 0 |
| 2018-12 | 17268 | 14516 | 2752 | 8423 | 0 | 4846 | 1334 | 875 | 0 |
| 2019-01 | 18629 | 15657 | 2972 | 8786 | 0 | 5126 | 1308 | 847 | 0 |
| 2019-02 | 17182 | 14592 | 2590 | 8356 | 0 | 4795 | 1145 | 813 | 0 |
| 2019-03 | 18043 | 15316 | 2727 | 8733 | 0 | 4821 | 1240 | 835 | 0 |
| 2019-04 | 18864 | 16104 | 2760 | 9125 | 0 | 5148 | 1391 | 893 | 0 |
| 2019-05 | 18941 | 16171 | 2770 | 8850 | 0 | 5158 | 1389 | 912 | 0 |
| 2019-06 | 17726 | 15239 | 2487 | 8204 | 0 | 4791 | 1330 | 818 | 0 |
| 2019-07 | 19255 | 16556 | 2699 | 9135 | 0 | 5173 | 1487 | 1093 | 0 |
| 2019-08 | 18710 | 16228 | 2482 | 8821 | 0 | 5014 | 1437 | 1189 | 0 |
| 2019-09 | 17662 | 15379 | 2283 | 8316 | 0 | 4737 | 1267 | 1102 | 0 |
| 2019-10 | 19367 | 16840 | 2527 | 9335 | 0 | 5106 | 1575 | 1003 | 0 |
| 2019-11 | 16592 | 14527 | 2065 | 8001 | 0 | 4451 | 1359 | 843 | 0 |
| 2019-12 | 19060 | 16749 | 2311 | 9088 | 0 | 5062 | 1536 | 953 | 0 |
| 2020-01 | 17404 | 15140 | 2264 | 8388 | 0 | 4798 | 1487 | 1000 | 0 |
| 2020-02 | 16806 | 14694 | 2112 | 8430 | 0 | 4624 | 1496 | 906 | 0 |
| 2020-03 | 20160 | 17665 | 2495 | 10120 | 0 | 5575 | 1843 | 1094 | 0 |
| 2020-04 | 19438 | 16987 | 2451 | 9478 | 0 | 5267 | 1753 | 1027 | 0 |
| 2020-05 | 18152 | 15915 | 2237 | 8903 | 0 | 4876 | 1692 | 948 | 0 |
| 2020-06 | 19323 | 16952 | 2371 | 9827 | 0 | 5158 | 1796 | 1019 | 0 |
| 2020-07 | 19036 | 16708 | 2328 | 9444 | 0 | 4989 | 1755 | 897 | 0 |
| 2020-08 | 17957 | 15813 | 2144 | 9009 | 0 | 4650 | 1666 | 819 | 0 |
| 2020-09 | 18475 | 16319 | 2156 | 9372 | 0 | 4765 | 1735 | 912 | 0 |
| 2020-10 | 18516 | 16407 | 2109 | 9289 | 0 | 4720 | 1755 | 968 | 0 |
| 2020-11 | 17547 | 15586 | 1961 | 8924 | 0 | 4594 | 1667 | 921 | 0 |
| 2020-12 | 19666 | 17537 | 2129 | 9832 | 0 | 5002 | 1969 | 932 | 0 |
| 2021-01 | 16685 | 14828 | 1857 | 8337 | 0 | 4316 | 1663 | 703 | 0 |
| 2021-02 | 16729 | 14854 | 1875 | 8396 | 0 | 4525 | 1762 | 757 | 0 |
| 2021-03 | 19436 | 17225 | 2211 | 10040 | 0 | 5387 | 2117 | 1136 | 0 |
| 2021-04 | 18175 | 16117 | 2058 | 9499 | 0 | 5083 | 1965 | 969 | 0 |
| 2021-05 | 17747 | 15813 | 1934 | 9006 | 0 | 4637 | 1863 | 938 | 0 |
| 2021-06 | 19925 | 17807 | 2118 | 10165 | 0 | 5050 | 2080 | 1068 | 0 |
| 2021-07 | 18810 | 16899 | 1911 | 9614 | 0 | 4768 | 1893 | 996 | 0 |
| 2021-08 | 19467 | 17526 | 1941 | 9973 | 0 | 4957 | 2067 | 1023 | 0 |
| 2021-09 | 19081 | 17190 | 1891 | 9814 | 0 | 4928 | 1979 | 1040 | 0 |
| 2021-10 | 17804 | 16100 | 1704 | 9358 | 0 | 4627 | 1910 | 816 | 0 |
| 2021-11 | 18414 | 16759 | 1655 | 9642 | 0 | 4792 | 1950 | 980 | 27 |
| 2021-12 | 19686 | 17930 | 1756 | 10333 | 0 | 4903 | 2089 | 1010 | 64 |
| 2022-01 | 18027 | 16399 | 1628 | 9465 | 0 | 4713 | 1936 | 913 | 103 |
| 2022-02 | 18012 | 16353 | 1659 | 9319 | 0 | 4638 | 1866 | 1017 | 117 |
| 2022-03 | 20799 | 18888 | 1911 | 10886 | 0 | 5267 | 2216 | 1117 | 138 |
| 2022-04 | 19080 | 17348 | 1732 | 9936 | 0 | 4710 | 2045 | 1028 | 186 |
| 2022-05 | 19577 | 17774 | 1803 | 10285 | 0 | 4974 | 2161 | 1130 | 219 |
| 2022-06 | 20478 | 18651 | 1827 | 10846 | 0 | 5153 | 2273 | 1220 | 212 |
| 2022-07 | 18843 | 17200 | 1643 | 9912 | 0 | 4609 | 2051 | 1033 | 264 |
| 2022-08 | 21015 | 19243 | 1772 | 11044 | 0 | 5182 | 2307 | 1212 | 334 |
| 2022-09 | 19663 | 17999 | 1664 | 10317 | 0 | 4829 | 2174 | 1027 | 320 |
| 2022-10 | 19029 | 17481 | 1548 | 9733 | 0 | 4501 | 2113 | 1078 | 308 |
| 2022-11 | 19391 | 17840 | 1551 | 10006 | 0 | 4737 | 2264 | 1028 | 369 |
| 2022-12 | 20046 | 18445 | 1601 | 10222 | 0 | 4813 | 2319 | 1042 | 376 |
| 2023-01 | 17500 | 16051 | 1449 | 9413 | 0 | 4563 | 2121 | 1039 | 384 |
| 2023-02 | 16190 | 14877 | 1313 | 9197 | 0 | 4318 | 2075 | 1075 | 432 |
| 2023-03 | 18418 | 16952 | 1466 | 10632 | 0 | 4908 | 2406 | 1216 | 501 |
| 2023-04 | 16173 | 14851 | 1322 | 9169 | 0 | 4170 | 2066 | 1070 | 461 |
| 2023-05 | 18007 | 16575 | 1432 | 10530 | 0 | 4887 | 2373 | 1256 | 550 |
| 2023-06 | 18075 | 16621 | 1454 | 10776 | 0 | 4806 | 2377 | 1213 | 566 |
| 2023-07 | 16635 | 15341 | 1294 | 9705 | 0 | 4340 | 2199 | 1131 | 537 |
| 2023-08 | 18599 | 17161 | 1438 | 10780 | 0 | 4706 | 2449 | 1242 | 605 |
| 2023-09 | 16438 | 15207 | 1231 | 9871 | 0 | 4307 | 2207 | 1036 | 587 |
| 2023-10 | 17808 | 16524 | 1284 | 10456 | 0 | 4312 | 2322 | 1164 | 618 |
| 2023-11 | 16908 | 15697 | 1211 | 10136 | 0 | 4384 | 2323 | 1159 | 665 |
| 2023-12 | 16623 | 15437 | 1186 | 10060 | 0 | 4193 | 2271 | 1042 | 769 |
| 2024-01 | 17194 | 15940 | 1254 | 10236 | 0 | 4547 | 2351 | 1154 | 764 |
| 2024-02 | 16324 | 15165 | 1159 | 9978 | 0 | 4262 | 2312 | 1055 | 814 |
| 2024-03 | 16204 | 15025 | 1179 | 10153 | 0 | 4283 | 2284 | 1257 | 795 |
| 2024-04 | 17043 | 15875 | 1168 | 10565 | 0 | 4430 | 2389 | 1308 | 855 |
| 2024-05 | 17490 | 16322 | 1168 | 11082 | 0 | 4459 | 2431 | 1371 | 919 |
| 2024-06 | 16191 | 15050 | 1141 | 9943 | 0 | 4028 | 2213 | 1233 | 869 |
| 2024-07 | 17524 | 16367 | 1157 | 11191 | 0 | 4439 | 2457 | 1467 | 913 |
| 2024-08 | 17203 | 16032 | 1171 | 10423 | 0 | 4298 | 2377 | 1339 | 773 |
| 2024-09 | 16148 | 15055 | 1093 | 10509 | 2950 | 4014 | 2261 | 1333 | 718 |
| 2024-10 | 17948 | 16816 | 1132 | 11921 | 5816 | 4666 | 2597 | 1532 | 735 |
| 2024-11 | 15921 | 14907 | 1014 | 11397 | 7188 | 4181 | 2316 | 1241 | 904 |

**Supplementary Table 2. FDA approval dates of CML Tyrosine kinase inhibitors (TKIs)**

| **Drug** | **FDA approval date** |
| --- | --- |
| Imatinib (Gleevec) | 05.10.2001 |
| Dasatinib (Sprycel) | 06.28.2006 |
| Nilotinib (Tasigna) | 10.29.2007 |
| Bosutinib (Bosulif) | 09.04.2012 |
| Ponatinib (Iclusig) | 12.14.2012 |
| Asciminib (Scemblix) | 10.29.2021 |

**Supplementary Table 3. Tyrosine kinase inhibitor generation prescriptions rates, IQVIA National Prescription Audit (2017 – 2024)^a^**

|  | **No. of average prescriptions per month (annual percent change)** | | |
| --- | --- | --- | --- |
| **Year** | **First Generation** | **Second Generation** | **Third Generation** |
| **2017** | 18704 | 15171 | 696 |
| **2018** | 17922 (-4.2%) | 15145 (-0.2%) | 824 (18.4%) |
| **2019** | 18336 (2.3%) | 15050 (-0.6%) | 942 (14.3%) |
| **2020** | 18540 (1.1%) | 15887 (5.6%) | 954 (1.3%) |
| **2021** | 18497 (-0.2%) | 16291 (2.5%) | 961 (0.7%) |
| **2022** | 19497 (5.4%) | 17152 (5.3%) | 1316 (37.0%) |
| **2023** | 17281 (-11.4%) | 16817 (-2.0%) | 1693 (28.7%) |
| **2024** | 16835 (-2.6%) | 17363 (3.2%) | 2123 (25.4%) |

^a^ Data for January 2017 – February 2017 and December 2024 not available

**Supplementary Table 4. Monthly Prescriptions of TKIs approved for treatment of CML by TKI generation and prescribing discipline**

| **date** | **Onco-logists**  **First Gene-ration** | **APP First Gene-ration** | **PCP/**  **Inter-**  **nists First Gene-ration** | **Other First Gene-ration** | **Onco-logists**  **Second Gene-ration** | **APP**  **Second Gene-ration** | **PCP/**  **Inter-**  **nists Second Gene-ration** | **Other Se-cond Gene-ration** | **Onco-logists**  **Third Gene-ration** | **APP Third Gene-ration** | **PCP/**  **Inter-**  **nists**  **Third Gene-ration** | **Other Third Gene-ration** |
| --- | --- | --- | --- | --- | --- | --- | --- | --- | --- | --- | --- | --- |
| 2017-03 | 14013 | 2057 | 1975 | 160 | 12356 | 1848 | 1247 | 116 | 509 | 118 | 45 | 11 |
| 2017-04 | 12604 | 1915 | 1785 | 141 | 11204 | 1679 | 1127 | 73 | 470 | 125 | 42 | 11 |
| 2017-05 | 13935 | 2184 | 2007 | 162 | 12432 | 2055 | 1244 | 95 | 477 | 128 | 47 | 10 |
| 2017-06 | 14586 | 2323 | 1993 | 162 | 12149 | 1978 | 1210 | 85 | 492 | 122 | 50 | 10 |
| 2017-07 | 14327 | 2322 | 1916 | 160 | 11391 | 1927 | 1065 | 69 | 459 | 124 | 40 | 7 |
| 2017-08 | 15287 | 2617 | 2059 | 160 | 12583 | 2169 | 1256 | 74 | 579 | 142 | 56 | 10 |
| 2017-09 | 13938 | 2369 | 1842 | 154 | 11257 | 1930 | 1121 | 50 | 504 | 108 | 47 | 8 |
| 2017-10 | 14920 | 2550 | 2014 | 151 | 12211 | 2088 | 1200 | 71 | 554 | 130 | 58 | 8 |
| 2017-11 | 14718 | 2423 | 1966 | 159 | 12164 | 2004 | 1205 | 59 | 499 | 144 | 49 | 7 |
| 2017-12 | 14430 | 2431 | 1980 | 143 | 11686 | 1999 | 1161 | 68 | 565 | 145 | 45 | 7 |
| 2018-01 | 14614 | 2524 | 2045 | 140 | 12207 | 2066 | 1253 | 78 | 509 | 125 | 49 | 8 |
| 2018-02 | 12572 | 2123 | 1709 | 120 | 11106 | 1819 | 1159 | 76 | 523 | 152 | 46 | 5 |
| 2018-03 | 14004 | 2464 | 1959 | 118 | 12358 | 2006 | 1266 | 74 | 611 | 195 | 54 | 5 |
| 2018-04 | 13462 | 2363 | 1808 | 133 | 11544 | 1965 | 1173 | 74 | 572 | 168 | 46 | 6 |
| 2018-05 | 14229 | 2456 | 1981 | 128 | 12621 | 2103 | 1282 | 85 | 596 | 178 | 53 | 12 |
| 2018-06 | 13418 | 2375 | 1771 | 137 | 11715 | 2008 | 1152 | 80 | 569 | 164 | 49 | 12 |
| 2018-07 | 13437 | 2423 | 1830 | 134 | 11890 | 2128 | 1193 | 71 | 587 | 164 | 56 | 8 |
| 2018-08 | 14213 | 2503 | 1933 | 135 | 12226 | 2199 | 1216 | 90 | 686 | 185 | 50 | 12 |
| 2018-09 | 12401 | 2197 | 1692 | 109 | 10669 | 1960 | 1016 | 85 | 561 | 152 | 50 | 8 |
| 2018-10 | 13924 | 2610 | 1890 | 124 | 12579 | 2334 | 1212 | 89 | 674 | 195 | 54 | 8 |
| 2018-11 | 13283 | 2441 | 1723 | 146 | 11624 | 2094 | 1109 | 88 | 610 | 185 | 56 | 5 |
| 2018-12 | 12968 | 2359 | 1770 | 171 | 11354 | 2113 | 1047 | 89 | 636 | 185 | 50 | 4 |
| 2019-01 | 13911 | 2649 | 1910 | 159 | 11700 | 2242 | 1194 | 84 | 595 | 193 | 54 | 5 |
| 2019-02 | 12888 | 2499 | 1658 | 137 | 10963 | 2154 | 1109 | 70 | 567 | 182 | 57 | 7 |
| 2019-03 | 13488 | 2666 | 1764 | 125 | 11331 | 2212 | 1160 | 91 | 587 | 189 | 52 | 7 |
| 2019-04 | 14148 | 2763 | 1810 | 143 | 12120 | 2318 | 1138 | 88 | 629 | 204 | 54 | 6 |
| 2019-05 | 14263 | 2757 | 1768 | 153 | 11900 | 2342 | 1082 | 73 | 644 | 202 | 56 | 10 |
| 2019-06 | 13224 | 2706 | 1616 | 180 | 11014 | 2209 | 1038 | 64 | 560 | 198 | 49 | 11 |
| 2019-07 | 14431 | 2890 | 1760 | 174 | 12211 | 2393 | 1113 | 78 | 769 | 259 | 56 | 9 |
| 2019-08 | 13985 | 2817 | 1728 | 180 | 11820 | 2294 | 1079 | 79 | 819 | 297 | 64 | 9 |
| 2019-09 | 13077 | 2722 | 1717 | 146 | 11059 | 2170 | 1019 | 72 | 774 | 269 | 52 | 7 |
| 2019-10 | 14403 | 2865 | 1917 | 182 | 12234 | 2504 | 1194 | 84 | 705 | 239 | 59 | 0 |
| 2019-11 | 12396 | 2509 | 1563 | 124 | 10592 | 2136 | 1008 | 75 | 595 | 202 | 45 | 1 |
| 2019-12 | 14245 | 2872 | 1787 | 156 | 11999 | 2504 | 1097 | 86 | 661 | 241 | 50 | 1 |
| 2020-01 | 12909 | 2737 | 1611 | 147 | 11096 | 2405 | 1110 | 62 | 697 | 243 | 59 | 1 |
| 2020-02 | 12556 | 2587 | 1524 | 139 | 11002 | 2454 | 1044 | 50 | 630 | 224 | 50 | 2 |
| 2020-03 | 14992 | 3233 | 1763 | 172 | 13204 | 2983 | 1269 | 82 | 758 | 266 | 67 | 3 |
| 2020-04 | 14512 | 3136 | 1604 | 186 | 12424 | 2804 | 1195 | 75 | 717 | 258 | 51 | 1 |
| 2020-05 | 13453 | 2919 | 1605 | 175 | 11653 | 2646 | 1127 | 45 | 658 | 239 | 49 | 2 |
| 2020-06 | 14358 | 3205 | 1603 | 157 | 12597 | 2862 | 1249 | 73 | 692 | 268 | 56 | 3 |
| 2020-07 | 14072 | 3201 | 1588 | 175 | 12176 | 2806 | 1139 | 67 | 621 | 210 | 63 | 3 |
| 2020-08 | 13274 | 3001 | 1545 | 137 | 11493 | 2674 | 1077 | 81 | 545 | 219 | 53 | 2 |
| 2020-09 | 13625 | 3158 | 1535 | 157 | 11818 | 2835 | 1145 | 74 | 620 | 225 | 61 | 6 |
| 2020-10 | 13670 | 3093 | 1607 | 146 | 11663 | 2846 | 1179 | 76 | 661 | 247 | 57 | 3 |
| 2020-11 | 12997 | 2909 | 1510 | 131 | 11232 | 2727 | 1155 | 71 | 616 | 237 | 65 | 3 |
| 2020-12 | 14584 | 3325 | 1626 | 131 | 12455 | 3050 | 1225 | 73 | 620 | 246 | 63 | 3 |
| 2021-01 | 12194 | 2908 | 1459 | 124 | 10619 | 2512 | 1113 | 72 | 458 | 187 | 54 | 4 |
| 2021-02 | 12233 | 2922 | 1453 | 121 | 10861 | 2624 | 1131 | 67 | 495 | 221 | 39 | 2 |
| 2021-03 | 14317 | 3346 | 1638 | 135 | 12876 | 3171 | 1393 | 104 | 765 | 301 | 67 | 3 |
| 2021-04 | 13307 | 3171 | 1572 | 125 | 12058 | 3098 | 1304 | 87 | 641 | 268 | 57 | 3 |
| 2021-05 | 12953 | 3126 | 1531 | 137 | 11386 | 2890 | 1162 | 68 | 629 | 242 | 63 | 4 |
| 2021-06 | 14494 | 3498 | 1749 | 184 | 12755 | 3129 | 1332 | 79 | 703 | 306 | 56 | 3 |
| 2021-07 | 13661 | 3309 | 1652 | 188 | 11938 | 3027 | 1248 | 62 | 669 | 262 | 62 | 3 |
| 2021-08 | 14141 | 3386 | 1770 | 170 | 12502 | 3124 | 1288 | 83 | 681 | 276 | 64 | 2 |
| 2021-09 | 13882 | 3404 | 1632 | 163 | 12373 | 3038 | 1244 | 66 | 695 | 276 | 66 | 3 |
| 2021-10 | 12930 | 3136 | 1578 | 160 | 11686 | 2947 | 1198 | 64 | 538 | 222 | 52 | 4 |
| 2021-11 | 13413 | 3252 | 1592 | 157 | 11997 | 3128 | 1196 | 63 | 683 | 262 | 58 | 4 |
| 2021-12 | 14385 | 3462 | 1656 | 183 | 12744 | 3248 | 1262 | 71 | 733 | 287 | 50 | 4 |
| 2022-01 | 13054 | 3221 | 1610 | 142 | 11773 | 3117 | 1177 | 47 | 711 | 248 | 53 | 4 |
| 2022-02 | 12982 | 3211 | 1656 | 163 | 11510 | 3065 | 1201 | 47 | 786 | 276 | 67 | 5 |
| 2022-03 | 15115 | 3754 | 1766 | 164 | 13557 | 3384 | 1376 | 52 | 847 | 334 | 67 | 7 |
| 2022-04 | 13766 | 3471 | 1671 | 172 | 12283 | 3126 | 1227 | 55 | 836 | 303 | 72 | 3 |
| 2022-05 | 14109 | 3561 | 1749 | 158 | 12856 | 3238 | 1279 | 47 | 942 | 329 | 72 | 6 |
| 2022-06 | 14766 | 3797 | 1743 | 172 | 13424 | 3414 | 1379 | 55 | 1010 | 338 | 76 | 8 |
| 2022-07 | 13435 | 3615 | 1604 | 189 | 12210 | 3089 | 1202 | 71 | 896 | 333 | 59 | 9 |
| 2022-08 | 14992 | 4013 | 1800 | 210 | 13513 | 3584 | 1368 | 68 | 1061 | 386 | 91 | 8 |
| 2022-09 | 14003 | 3749 | 1733 | 178 | 12677 | 3320 | 1251 | 72 | 964 | 306 | 71 | 6 |
| 2022-10 | 13579 | 3647 | 1633 | 170 | 11909 | 3204 | 1154 | 80 | 996 | 311 | 72 | 7 |
| 2022-11 | 13880 | 3734 | 1615 | 162 | 12488 | 3291 | 1155 | 73 | 1000 | 305 | 85 | 7 |
| 2022-12 | 14263 | 3923 | 1712 | 148 | 12701 | 3440 | 1151 | 62 | 1007 | 325 | 82 | 4 |
| 2023-01 | 12458 | 3436 | 1477 | 129 | 11716 | 3195 | 1143 | 43 | 970 | 360 | 84 | 9 |
| 2023-02 | 11498 | 3280 | 1287 | 125 | 11361 | 3100 | 1070 | 59 | 1040 | 372 | 90 | 5 |
| 2023-03 | 13023 | 3645 | 1623 | 127 | 13018 | 3675 | 1201 | 52 | 1164 | 456 | 93 | 4 |
| 2023-04 | 11336 | 3246 | 1486 | 105 | 11173 | 3140 | 1040 | 52 | 1043 | 404 | 83 | 1 |
| 2023-05 | 12855 | 3595 | 1444 | 113 | 12934 | 3622 | 1185 | 49 | 1218 | 489 | 92 | 7 |
| 2023-06 | 12803 | 3685 | 1478 | 109 | 12898 | 3788 | 1229 | 44 | 1209 | 455 | 113 | 2 |
| 2023-07 | 11829 | 3323 | 1351 | 132 | 11784 | 3372 | 1039 | 49 | 1100 | 465 | 101 | 2 |
| 2023-08 | 13103 | 3867 | 1483 | 146 | 12891 | 3835 | 1156 | 53 | 1220 | 519 | 106 | 2 |
| 2023-09 | 11580 | 3457 | 1285 | 116 | 11864 | 3421 | 1040 | 60 | 1057 | 467 | 96 | 3 |
| 2023-10 | 12662 | 3662 | 1384 | 100 | 12252 | 3670 | 1114 | 54 | 1141 | 529 | 108 | 4 |
| 2023-11 | 11855 | 3614 | 1348 | 91 | 12119 | 3608 | 1064 | 52 | 1195 | 522 | 103 | 4 |
| 2023-12 | 11588 | 3545 | 1385 | 105 | 11832 | 3582 | 1062 | 48 | 1195 | 501 | 112 | 3 |
| 2024-01 | 12115 | 3548 | 1412 | 119 | 12297 | 3743 | 1052 | 42 | 1271 | 537 | 108 | 2 |
| 2024-02 | 11553 | 3379 | 1293 | 99 | 11889 | 3628 | 996 | 39 | 1230 | 522 | 112 | 5 |
| 2024-03 | 11284 | 3421 | 1370 | 129 | 12016 | 3636 | 1028 | 40 | 1384 | 547 | 119 | 2 |
| 2024-04 | 12003 | 3566 | 1356 | 118 | 12341 | 3919 | 1079 | 45 | 1419 | 601 | 139 | 4 |
| 2024-05 | 12411 | 3630 | 1335 | 114 | 12767 | 4073 | 1093 | 39 | 1502 | 648 | 140 | 0 |
| 2024-06 | 11440 | 3301 | 1327 | 123 | 11444 | 3652 | 1041 | 47 | 1404 | 588 | 110 | 0 |
| 2024-07 | 12230 | 3761 | 1376 | 157 | 12630 | 4234 | 1168 | 55 | 1564 | 680 | 132 | 4 |
| 2024-08 | 11991 | 3702 | 1346 | 164 | 11928 | 4056 | 1066 | 48 | 1383 | 618 | 108 | 3 |
| 2024-09 | 11124 | 3489 | 1394 | 141 | 11853 | 3819 | 1061 | 51 | 1337 | 603 | 107 | 4 |
| 2024-10 | 12329 | 4032 | 1464 | 123 | 13470 | 4369 | 1285 | 60 | 1474 | 674 | 116 | 3 |
| 2024-11 | 11044 | 3452 | 1312 | 113 | 12507 | 4188 | 1137 | 62 | 1405 | 629 | 108 | 3 |

**Supplementary Table 5. Monthly Online Searches (per 10 million) for TKIs approved for treatment of CML**

| **date** | **Imatinib** | **Dasatinib** | **Nilotinib** | **Bosutinib** | **Ponatinib** | **Asciminib** |
| --- | --- | --- | --- | --- | --- | --- |
| 2017-03 | 21.33401 | 7.465805 | 6.461311 | 0.859075 | 1.645198 | 0 |
| 2017-04 | 16.17842 | 6.319017 | 5.147475 | 1.237868 | 1.570007 | 0 |
| 2017-05 | 16.93087 | 7.380967 | 5.158555 | 1.382363 | 1.509406 | 0 |
| 2017-06 | 16.86913 | 6.028122 | 4.6335 | 1.403622 | 1.524249 | 0 |
| 2017-07 | 16.30392 | 7.04985 | 4.572124 | 1.358446 | 1.296082 | 0 |
| 2017-08 | 15.69578 | 7.946013 | 5.225898 | 1.982272 | 1.870885 | 0 |
| 2017-09 | 16.15027 | 7.053627 | 5.224994 | 1.314945 | 0.823392 | 0 |
| 2017-10 | 17.05527 | 7.608203 | 5.876046 | 1.566775 | 1.969469 | 0 |
| 2017-11 | 18.58682 | 7.669576 | 5.407481 | 1.507002 | 1.622841 | 0 |
| 2017-12 | 14.33004 | 6.757185 | 5.072451 | 1.295784 | 1.535899 | 0 |
| 2018-01 | 17.21728 | 8.172381 | 5.9546 | 1.818059 | 2.062152 | 0 |
| 2018-02 | 19.26631 | 7.760803 | 6.110651 | 1.555614 | 2.385352 | 0 |
| 2018-03 | 18.25768 | 8.021474 | 6.31681 | 1.857861 | 2.096235 | 0 |
| 2018-04 | 17.88353 | 8.211886 | 6.332164 | 1.508129 | 1.817208 | 0 |
| 2018-05 | 16.81449 | 6.722041 | 6.872287 | 1.753585 | 1.931975 | 0 |
| 2018-06 | 16.17225 | 7.175567 | 5.183966 | 1.640124 | 1.920961 | 0 |
| 2018-07 | 17.48551 | 8.888226 | 6.052377 | 1.529125 | 1.937464 | 0 |
| 2018-08 | 15.85031 | 8.344959 | 8.266418 | 1.935175 | 2.015706 | 0 |
| 2018-09 | 15.90011 | 7.333036 | 8.041246 | 1.16971 | 1.63226 | 0 |
| 2018-10 | 17.01907 | 8.422636 | 6.575317 | 1.416427 | 1.600242 | 0 |
| 2018-11 | 17.27518 | 6.864531 | 5.235419 | 1.930691 | 1.544846 | 0 |
| 2018-12 | 14.23137 | 7.358781 | 5.763273 | 1.551351 | 1.20656 | 0 |
| 2019-01 | 17.57579 | 10.31532 | 4.905872 | 1.671002 | 1.728801 | 0 |
| 2019-02 | 18.26795 | 8.747279 | 5.041408 | 1.736419 | 1.706058 | 0 |
| 2019-03 | 16.45546 | 8.910047 | 5.277345 | 2.229822 | 2.149601 | 0 |
| 2019-04 | 17.31932 | 8.486315 | 5.496939 | 1.992716 | 1.598766 | 0 |
| 2019-05 | 14.64344 | 8.103301 | 5.682489 | 1.739182 | 1.766233 | 0 |
| 2019-06 | 14.80231 | 7.772867 | 4.83221 | 1.549312 | 1.561786 | 0 |
| 2019-07 | 14.60822 | 8.031633 | 5.211986 | 1.838899 | 1.594191 | 0 |
| 2019-08 | 15.24001 | 8.11673 | 4.754603 | 1.446255 | 1.871563 | 0 |
| 2019-09 | 16.39642 | 7.91968 | 5.248345 | 1.307474 | 1.483583 | 0 |
| 2019-10 | 15.95972 | 9.222347 | 5.106006 | 1.745551 | 1.796911 | 0 |
| 2019-11 | 16.0702 | 7.415795 | 4.668575 | 1.444435 | 2.097024 | 0 |
| 2019-12 | 14.91959 | 7.415886 | 5.963496 | 1.778651 | 1.71955 | 0.493091 |
| 2020-01 | 18.22496 | 9.340276 | 5.648082 | 1.73574 | 1.722648 | 0 |
| 2020-02 | 16.41208 | 9.765078 | 4.407433 | 1.378783 | 1.922719 | 0.409646 |
| 2020-03 | 14.14983 | 7.969849 | 4.550326 | 1.803506 | 1.524875 | 0 |
| 2020-04 | 13.05115 | 7.39448 | 3.577245 | 1.727327 | 1.751102 | 0.361132 |
| 2020-05 | 13.38525 | 7.544197 | 3.332754 | 1.467467 | 1.528712 | 0.286524 |
| 2020-06 | 12.39692 | 7.175855 | 4.246087 | 1.687929 | 1.901504 | 0 |
| 2020-07 | 13.43117 | 8.349704 | 4.1132 | 1.966335 | 1.492046 | 0 |
| 2020-08 | 14.4124 | 8.553979 | 5.20923 | 1.731477 | 2.103271 | 0 |
| 2020-09 | 13.76158 | 8.034469 | 5.175317 | 1.928701 | 1.879874 | 0 |
| 2020-10 | 14.32935 | 8.038616 | 4.438536 | 2.089228 | 1.875468 | 0 |
| 2020-11 | 13.1489 | 8.059701 | 4.505002 | 1.39393 | 1.433243 | 0.36533 |
| 2020-12 | 12.51787 | 7.214465 | 4.429386 | 1.424522 | 1.310929 | 0.422872 |
| 2021-01 | 12.74722 | 7.14962 | 4.366022 | 1.771323 | 1.308704 | 0.409044 |
| 2021-02 | 14.19609 | 10.0558 | 4.511793 | 1.649044 | 1.309699 | 0.471062 |
| 2021-03 | 13.54857 | 8.783511 | 4.368296 | 1.652594 | 1.410761 | 0.34732 |
| 2021-04 | 14.20785 | 8.820571 | 4.74677 | 1.987984 | 1.984734 | 0.47307 |
| 2021-05 | 13.91418 | 8.780053 | 4.723608 | 2.024714 | 1.670185 | 0.34633 |
| 2021-06 | 13.29662 | 8.117657 | 4.504762 | 1.895423 | 1.435897 | 0 |
| 2021-07 | 12.52604 | 8.93868 | 4.46525 | 1.876807 | 1.787736 | 0.383123 |
| 2021-08 | 13.80575 | 8.349958 | 4.118814 | 1.323634 | 1.995367 | 0.517579 |
| 2021-09 | 13.93997 | 9.706595 | 4.066077 | 1.566178 | 1.512763 | 0.502637 |
| 2021-10 | 13.70649 | 8.666619 | 4.808486 | 2.05406 | 1.640941 | 0.779544 |
| 2021-11 | 13.36099 | 9.659909 | 5.392567 | 1.913909 | 1.794142 | 3.463725 |
| 2021-12 | 11.1147 | 8.242504 | 4.509206 | 1.682371 | 1.847455 | 2.171709 |
| 2022-01 | 19.7681 | 11.25089 | 4.128404 | 2.27242 | 2.283845 | 2.042048 |
| 2022-02 | 18.30857 | 10.26905 | 4.214787 | 1.942081 | 2.484516 | 2.143331 |
| 2022-03 | 15.18379 | 8.637926 | 4.38517 | 2.085054 | 2.129582 | 1.729574 |
| 2022-04 | 14.87417 | 9.264974 | 3.774734 | 2.081373 | 2.310537 | 1.587721 |
| 2022-05 | 14.71089 | 7.812127 | 3.867924 | 1.739888 | 1.678497 | 1.921953 |
| 2022-06 | 16.91528 | 9.272039 | 3.874097 | 1.673847 | 1.744288 | 1.955165 |
| 2022-07 | 13.5928 | 8.995486 | 4.350538 | 1.882585 | 2.168187 | 1.626361 |
| 2022-08 | 16.39149 | 8.782787 | 4.726103 | 1.689456 | 1.967225 | 1.982257 |
| 2022-09 | 14.31225 | 8.366163 | 4.160669 | 1.861942 | 1.631293 | 1.609228 |
| 2022-10 | 13.18658 | 9.218035 | 3.893189 | 1.739914 | 1.608764 | 1.540974 |
| 2022-11 | 13.78112 | 8.466303 | 4.529841 | 1.726478 | 1.813279 | 1.275855 |
| 2022-12 | 12.52343 | 8.229643 | 3.65856 | 1.571327 | 1.557053 | 1.46067 |
| 2023-01 | 12.92215 | 8.575064 | 4.25686 | 2.392192 | 2.095 | 1.535623 |
| 2023-02 | 14.75099 | 9.139441 | 3.632463 | 1.957587 | 1.900613 | 1.583629 |
| 2023-03 | 14.95072 | 8.696972 | 4.07783 | 2.02351 | 2.510202 | 1.222345 |
| 2023-04 | 14.04647 | 8.354361 | 3.738666 | 1.881362 | 2.22102 | 1.80497 |
| 2023-05 | 14.85676 | 8.513172 | 3.587031 | 1.66102 | 2.187371 | 1.438132 |
| 2023-06 | 13.73467 | 8.749563 | 3.18568 | 2.059653 | 2.088188 | 1.436874 |
| 2023-07 | 12.25834 | 8.249868 | 3.10613 | 2.00581 | 2.173571 | 1.714948 |
| 2023-08 | 12.26258 | 8.060705 | 3.204652 | 1.876465 | 1.745238 | 1.465368 |
| 2023-09 | 13.49135 | 7.704222 | 3.282829 | 2.316292 | 2.047751 | 1.469386 |
| 2023-10 | 13.67216 | 8.261032 | 3.030533 | 2.3153 | 2.503026 | 1.433039 |
| 2023-11 | 13.7728 | 8.207298 | 3.808249 | 2.352958 | 1.862021 | 1.546411 |
| 2023-12 | 11.64462 | 8.542798 | 3.221948 | 1.81398 | 1.830465 | 1.535603 |
| 2024-01 | 13.64488 | 9.628376 | 3.829113 | 2.327522 | 2.046204 | 2.141351 |
| 2024-02 | 14.04987 | 9.942874 | 3.233782 | 1.832228 | 2.78371 | 2.291145 |
| 2024-03 | 12.63801 | 8.802407 | 3.213483 | 1.842005 | 2.403705 | 2.035135 |
| 2024-04 | 13.68179 | 8.550238 | 3.88576 | 1.973935 | 2.061707 | 2.003441 |
| 2024-05 | 12.90672 | 8.402436 | 3.247792 | 1.815294 | 2.003906 | 2.428525 |
| 2024-06 | 13.88493 | 7.836424 | 3.522892 | 2.09168 | 1.791115 | 2.668447 |
| 2024-07 | 12.04401 | 7.347795 | 4.161785 | 2.434893 | 2.23745 | 2.763455 |
| 2024-08 | 13.46926 | 8.061775 | 3.729123 | 1.463028 | 2.174188 | 3.034345 |
| 2024-09 | 13.2469 | 9.061228 | 2.924595 | 1.91476 | 2.589862 | 2.233479 |
| 2024-10 | 13.01358 | 9.048828 | 3.539069 | 2.327325 | 2.226032 | 3.005 |
| 2024-11 | 12.50576 | 8.69379 | 3.688872 | 1.704417 | 2.481828 | 3.433622 |

**Supplementary Table 6. Online searches for Tyrosine kinase inhibitors (TKIs) aggregated by drug generation, Google Trends (2017- 2024)^a^**

|  | **No. of online searches per 10 million (annual percent change)** | | |
| --- | --- | --- | --- |
|  | **First Generation** | **Second Generation** | **Third Generation** |
| **2017** | 16.9 | 13.8 | 1.5 |
| **2018** | 16.9 (0.0%) | 15.8 (14.5%) | 1.8 (20.1%) |
| **2019** | 16.0 (-5.5%) | 15.3 (-3.4%) | 1.8 (-2.6%) |
| **2020** | 14.1 (-12.0%) | 14.3 (-6.4%) | 1.9 (3.4%) |
| **2021** | 13.4 (-5.2%) | 15.1 (5.7%) | 2.5 (32.6%) |
| **2022** | 15.3 (14.5%) | 15.0 (-0.5%) | 3.7 (49.7%) |
| **2023** | 13.5 (-11.5%) | 14.0 (-7.0%) | 3.6 (-2.0%) |
| **2024** | 13.2 (-2.5%) | 14.2 (1.4%) | 4.8 (33.0%) |

^a^ Data for January 2017 – February 2017 and December 2024 not available
